# Boosting wheat yield, profitability and NUE with prilled and nano urea in conservation tillage

**DOI:** 10.1038/s41598-023-44879-w

**Published:** 2023-10-23

**Authors:** Nitesh Kumar, S. C. Tripathi, D. B. Yadav, Shiv Ram Samota, Karnam Venkatesh, Sindhu Sareen, Gyanendra Singh

**Affiliations:** 1 ICAR-Indian Institute of Wheat and Barley Research, Agarsain Marg, P.B. No. 158, Karnal, Haryana 132 001 India; 2 CCS HAU, RRS, Bawal, Haryana 123 501 India; 3 ICAR-Indian Institute of Millets Research, Rajendranagar, Hyderabad, Telangana 500 030 India

**Keywords:** Physiology, Plant sciences

## Abstract

Rice–wheat production in the Indo-gangetic plains (IGPs) of India faces major concerns such as depleting resources, rice residue burning, excessive fertilizer use, and decreasing nitrogen use efficiency. These issues threaten sustainable crop production in the future. Therefore, a field study was conducted during the winter seasons of 2020–21 and 2021–22 to evaluate the effect of combined conventional and nano fertilizers on nitrogen application just before or after irrigation to improve wheat productivity, profitability and NUE under conservation tillage. The study evaluated eight treatment combinations of nitrogen application through conventionally applied urea (46% N) and foliar applied nano urea (4% N) under zero tillage with rice residue retention. Results revealed that growth, physiological indices, yield, and quality parameters were enhanced with the application of 150 kg N/ha in three equal splits as basal and just before 1st and 2nd irrigation alone (T2) or along with a spray of nano urea (T5) compared to other treatments. T5 recorded 7.2%, 8.5%, and 7.8% more plant dry matter, number of tillers, and grain yield, respectively, over the conventional practice of applying 150 kg N/ha in three equal splits as basal and 7–10 days after 1st and 2nd irrigation (T3, farmers practice). Although, T2 showed similar results to T5, T5 recorded significantly higher gross ($2542/ha) and net returns ($1279/ha) than the other treatments. However, the benefit–cost ratio of T2 and T5 was same (2.01). A significant and positive correlation coefficient between grain yield and physiological parameters such as CCI and NDVI confirmed that increasing the nitrogen dose enhanced the chlorophyll content, greenness, and plant vigor. Based on the results, it can be concluded that applying 150 kg N/ha in three equal splits as basal and just before 1st and 2nd irrigation under conservation agriculture, along with a single spray of nano urea (4% N) at 60–65 days after sowing, can improve growth, yield attributes, wheat yield, and NUE compared to farmers practice (T3) in India.

## Introduction

India contributes approximately 13.9% (106.84 mt; 4th estimate by GoI, 2021–22) of the global wheat production, which is 770.3 mt^1^, ranking second after China. However, India's wheat productivity is lower than the global wheat productivity. The Indo-Gangetic Plains (IGPs) of India, particularly Punjab and Haryana, are known as the grain bowl of the country where the rice–wheat cropping system is dominant. The productivity of wheat depends on proper monetary and non-monetary inputs, tillage methods, and better agronomic management practices. Among tillage options, conventional tillage involves intensive soil disturbance with multiple passes of tillage equipment to accomplish fine land preparation for seed sowing, which is a waste of energy resources, lacks sustainability and also results in environmental hazards^2^. These tillage operations require considerable amount of time and in many cases, it defers the wheat sowing especially in rice–wheat system where rice harvesting is delayed. The delay in wheat sowing beyond 15th November can reduce the yield by 26.8 kg/day/ha^3^. These concerns prompt the farmer to burn the rice residue for its rapid management and timely seeding of wheat crop in rice–wheat system especially in Punjab and Haryana states.

India produces more than 686 million tonnes of crop residue every year^4,5^. In the IGPs of India, burning of crop residue, particularly of rice, is a problem that leads to degradation of soil health and environmental pollution^6,7^. To counter these negative impacts, conservation tillage gaining popularity among farmers due to its ability to address the challenges of residue management and timely sowing of wheat crop. CA has been suggested as a potential agricultural strategy due to its capacity to store carbon and slow down global warming^8,9^. Its practices allow timely sowing of crops, minimize cost, improve soil aggregate stability, and protect the environment in the long term. This practice is economically feasible, environmentally sound, socially acceptable and sustainable^10^. Happy Seeder-based wheat sowing is more profitable (10–20%) and has scalable residue management practices over burning^6^. In conservation tillage, wheat is directly seeded under no-till condition in the presence of previous crop residue using suitable machinery such as zero-till drill, happy seeder, strip-till drill, etc. The conservation agriculture has the potential to manage the 66.7% of total crop residue produced in India annually^5^.

Among monetary inputs, nutrient management is crucial for achieving self-sufficiency in food grain production, but blind use of fertilizers has resulted in higher nutrient consumption and lowered profits^11^. Conventional fertilizer uses efficiencies have not improved significantly over the years, with N, P, and K efficiencies hardly exceeding 30–35%, 18–20%, and 35–40%, respectively. The extent of multi-nutrient deficiencies is alarmingly increasing year by year, closely associated with a crop loss of nearly 25–30%. The extent of nutrient deficiencies in the country is high, with N, P, K, S, Zn, and B deficiencies ranging from 33 to 90%^12^. By 2025, the country will need to procure approximately 40–45 mt of nutrients to meet the demand^13^. Among all the essential nutrients, nitrogen is the primary and most vital one. In the sandy loam soils of the Indo-Gangetic Plains (IGPs) in India, nitrogen is typically found in low quantities, usually below 280 kg/ha. This necessitates the introduction of external fertilizer sources to supply nitrogen to the soil. Historically, nitrogen has predominantly been provided through urea in the IGPs of India. However, the imbalanced and excessive use of conventional urea (containing 46% nitrogen) with the sole aim of increasing productivity and profitability has led to a decline in the soil's response to fertilizer application. The nitrogen use efficiency of urea is typically low, ranging from 20 to 50% in most of the soils^14^. Nitrogen use efficiency decreases as nitrogen doses increase due to its susceptibility to various losses such as leaching, denitrification, and volatilization^15,16^. According to reports from the Fertilizer Association of India, the total urea consumption in India in the same period amounted to 34.18 million metric tons. Of this total, 25.08 million metric tons were domestically produced, while 9.14 million metric tons were imported. During the fiscal year 2021–22, the central government provided a subsidy of 75,902 crore rupees for urea, which includes both indigenous and imported varieties. The central government also implemented a nutrient-based subsidy during the winter cropping season of 2022–23, offering a subsidy of ₹98.02 per kilogram for nitrogen. This subsidy significantly exceeds the market selling price of nitrogen for farmers.

In recent times, nanotechnology has gained prominence, with a particular focus on the use of nano-fertilizers in order to enhance the use efficiency. These fertilizers can enhance nutrient use efficiency by exploiting the unique properties of particles ranging from 1 to 100 nm in size^17^. Nano-fertilizers have desirable features such as high solubility, stability, effectiveness, time-controlled release, targeted activity, low eco-toxicity, and easy modes of delivery and disposal^18^. A recent review on nano-fertilizers confirmed their high solubility, consistency, effectiveness, time-controlled discharge, targeted activity, and safe, simple delivery and disposal methods^19^. Crop residue recycling through the adoption of CAs can also be quite helpful as it contains a significant amount of nutrients.

In Indian farming practices, N application in wheat is recommended 7–10 days after irrigation, whereas under CA, there is an urgent need to apply just before irrigation. Hence, CA coupled with nano-fertilizers can play a greater role in improving crop production with environmental safety, ecological sustainability, and economic stability. Fertilizer nutrient use efficiency, particularly N, in crop production can be enhanced with the effective use of NFs. Nano-fertilizers improve crop growth and yield up to optimum applied doses and concentrations, but they also have an inhibitory effect on crop plants if the concentration is higher than the optimum, which might reduce growth and yield of the crop. There is a paucity of information on these aspects under Indian conditions, and systematic investigations are required.

The experiment tested the hypothesis that rice residue management by CA and judicious N management with the use of conventional and nano-fertilizers of nitrogen enhance the nitrogen use efficiency, productivity, and profitability of wheat.

## Material and methods

### Study site

**Figure 1:**
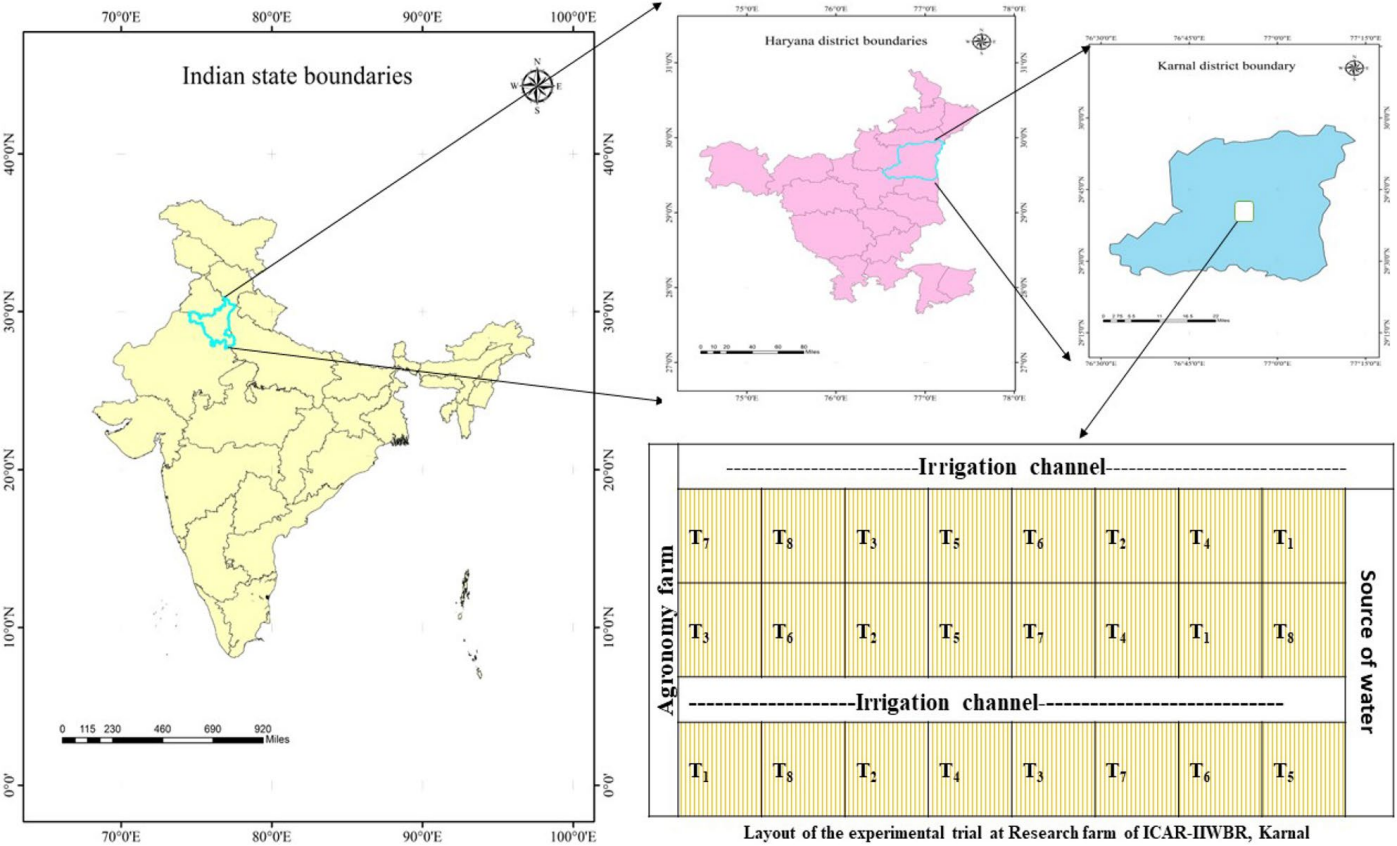
Location and layout of experimental trial at resource management farm of ICAR-IIWBR, Karnal (The map was extracted by using shapefile tool in QGIS 3.8.2 software).

A 2-year study was conducted at the ICAR-Indian Institute of Wheat and Barley Research’s Research Farm in Karnal (Fig. 1), Haryana, India (29° 43′ N; 76° 58′ E, altitude 245 m above mean sea level) from 2020 to 2022.

### Weather conditions

**Figure 2:**
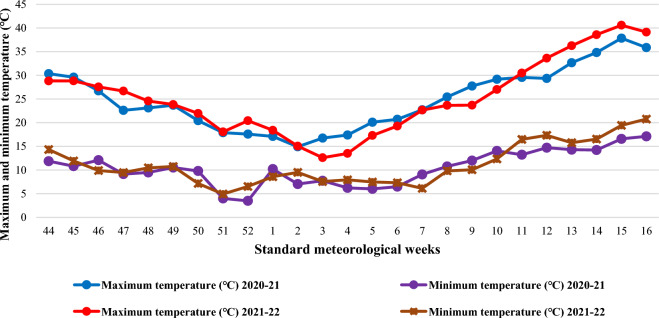
Mean weekly maximum and minimum temperature during the crop growing seasons (2020–21 & 2021–22).

**Figure 3:**
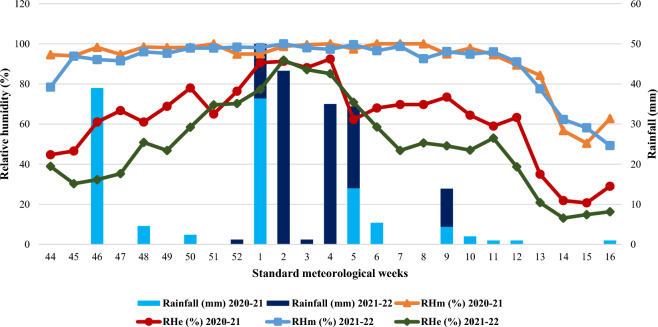
Mean weekly relative humidity and total rainfall during the crop growing seasons (2020–21 & 2021–22).

The experimental site is situated in the sub-tropical and semi-arid climate of Haryana, India. The region experiences cold winters from November to January, followed by mild temperatures in February and March. Mean weekly meteorological observations were taken during the cropping season from November 2020 to April 2021, and again from November 2021 to April 2022, and were recorded at the meteorological observatory of ICAR-Central Soil Salinity Research Institute in Karnal. These observations are presented in Figs. 2 and 3.

Weekly maximum and minimum temperatures ranged from 14.9 ℃ to 37.8 ℃ and from 3.5 ℃ to 17.1 ℃ in 2020–21 and from 12.6 ℃ to 40.6 ℃ and from 4.9 ℃ to 19.4 ℃ in 2021–22, respectively (Fig. 2). During the year 2021–22, the maximum and minimum temperature during the post-anthesis stage was higher than the previous year. The weekly averages of morning and evening relative humidity varied between 50.4 to 100.0 and 20.7 to 92.4 percent in 2020–21 and 49.3 to 100.0 and 13.1 to 91.9 percent in 2021–22, respectively (Fig. 3).

The total rainfall received during the crop seasons was 124.2 mm and 111.2 mm during 2020–21 and 2021–22, respectively (Fig. 3). With the exception of an increase in the maximum (average of 11th to 16th meteorological weeks was approximately 3.1 ℃) and minimum (average of 11th to 16th meteorological weeks was approximately 2.7 ℃) temperatures, which caused terminal heat stress during the grain development stage in second year of study i.e. 2021–22. While the other weather parameters were conducive to the growth and development of wheat.

### Experimental design, treatments and crop management

Prior to commencing the present experiment, the field was cultivated with transplanted rice in the summer of 2020 using traditional farmers’ practices to ensure soil fertility homogeneity across the experimental field. The initial physico-chemical properties of the soil at depths of 0–5, 5–10, and 10–15 cm were analyzed before the start of the experiment. The soil of the experimental site lies in inceptisol order which was sandy loam in texture, with sand content of 62.4%, silt content of 27.5%, and clay content of 10.1%. The values of other soil properties, such as pH1:2.5, electrical conductivity1:2.5, organic carbon, and available N, P, and K, are given in Table 1.

**Table 1:** Chemical characteristics of soil prior to the experimentation.

SN	Soil properties	Values of soil parameters		
	Chemical analysis	0–5 cm	5–10 cm	10–15 cm
1.	pH	7.93	8.00	8.12
2.	EC (dS/m)	0.187	0.165	0.147
3.	Organic carbon (%)	0.62	0.59	0.55
4.	Available nitrogen (kg/ha)	173.2	164.6	156.4
5.	Available phosphorus (kg/ha)	17.62	15.84	14.74
6.	Available potassium (kg/ha)	182.4	162.1	152.2

The field experiment was conducted during two winter seasons in 2020–21 and 2021–22 to evaluate the effect of different nano urea sprays on wheat and compare their outcome with recommended conventional urea in conservation tillage, with the goal of reducing dependence on conventional nitrogen fertilizers. Prior to the experimentation, the cropping sequence practiced in the experimental plot was puddled transplanted rice -conventional tillage wheat. The experimental design was a randomized block with eight treatments and three replications, using gross plot areas of 8.0 m × 2.0 m and net plot areas of 6.0 m × 1.6 m. Glyphosate herbicide was sprayed to remove weeds prior to sowing. Wheat variety “DBW-187” was line-sown at a rate of 125 kg/ha with 20 cm spacing, using a zero drill Kamboj happy seeder and leaving residue from the previous crop (puddled transplanted rice @ 6 t/ha). The sowing date was 1st November 2020 and 9th November 2021 for 2020–21 and 2021–22, respectively.

The experimental plots received different doses of nitrogen (with the recommended dose at 150 kg/ha) through prilled urea (46% N) as per the treatments outlined in Table 2. A full dose of phosphorus (60 kg/ha) and potassium (40 kg/ha) were applied at the time of sowing in all treatments by using single super phosphate (16% P_2_O_5_) and muriate of potash (60% K_2_O), respectively. Nano urea (an IFFCO product) with 40,000 ppm of nitrogen was sprayed as per the treatment. Nano nitrogen was applied at a rate of 4 ml of nano urea/liter of water with a product dose of 1250 ml/ha. Foliar spray of nano urea was carried out using knapsack power sprayer having two nozzle spray boom after proper calibration and in synchrony with seed drilling. The detailed timing and weather conditions are given in Table 3. Other agronomic practices were carried out as per the recommendation of ICAR-IIWBR, Karnal. In 2020–21, six irrigations were applied at critical growth stages of wheat, while in 2021–22, an additional irrigation was applied at the grain filling stage due to an increase in temperature during the later stages. Glyphosate 41% SL @ 2.5 l/ha was sprayed a week before sowing as chemical plowing for weed management. After sowing, post-emergence applications of clodinafop 15% WP @ 60 g a.i./ha and metsulfuron methyl 20% WP @ 4 g *a.i*./ha were used for proper weed management. Termite attack was controlled by spraying chlorophyrifos 20% EC @ 2.5 l/ha, and rust was managed by spraying Propiconazole 25% EC @500 ml/ha just before flowering. At maturity, the crop was manually harvested and sun-dried. Mechanical threshing was done using *Hadamba* wheat thresher. The threshed grains were weighed and converted into kilograms per hectare for the estimation of yield.

**Table 2:** Performance of nano-nitrogen fertilizer under conservation tillage (rice residue retention condition).

	0 DAS	20–25 DAS	40–45 DAS	60–65 DAS	80–85 DAS
T_1_	Absolute control (no nitrogen)				
T_2_	1/3rd N as basal + full PK	1/3rd N each just before 1st and 2nd irrigation (BI)			
T_3_	1/3rd N as basal + full PK	1/3rd N each, 7–10 days after 1st and 2nd irrigation (AI) (Farmers practice)			
T_4_	1/3rd N as basal + full PK	1/3rd N just before 1st irrigation	Spray of Nano N*(4%)		
T_5_	1/3rd N as basal + full PK	1/3rd N each just before 1st and 2nd irrigation (BI)		Spray of Nano N*	
T_6_	1/3rd N as basal + full PK	1/3rd N just before 1st irrigation	Spray of Nano N*	Spray of Nano N*	
T_7_	1/3rd N as basal + full PK	Spray of Nano N*	Spray of Nano N*	Spray of Nano N*	
T_8_	1/3rd N as basal + full PK	Spray of Nano N*	Spray of Nano N*	Spray of Nano N*	Spray of Nano N*

*DAS* days after sowing.

*Nano N spray through Nano urea @4 ml/l with 1250 ml/ha of product dose, T_3_: Farmers’ practice.

**Table 3:** Details of the weather conditions during foliar spray regime.

Foliar spray	Maximum temperature (℃)	Minimum temperature (℃)	Relative humidity at evening (%)
22nd November 2020	22.6	9.1	66.7
14th December 2020	20.4	9.8	78.0
5th January 2021	17.1	10.2	90.6
27th January 2021	17.4	6.2	92.4
1st December 2021	24.6	10.5	50.9
22nd December 2021	18.1	4.9	69.6
12th January 2022	15.1	9.5	91.9
3rd February 2022	17.3	7.5	70.9

### Observations recorded

Data were collected on plant height, dry matter accumulation, leaf area index, grain yield, number of effective tillers per m^2^, thousand grains weight (TGW), leaf chlorophyll content or greenness, and spectral reflectance of leaves or NDVI values. Plant height and dry matter accumulation were measured at harvest. The leaf area index was measured by using the “Sun Scan Canopy Analyser” from three sites in each plot. The grain yield was calculated from the net plot area and then converted into kg/ha. The number of effective tillers per meter row length was counted at two places in each plot and converted to per m^2^. The TGW was calculated by taking random grain samples and counting them using a Contador electronic seed counter (Pfeuffer, Germany) and weighing them.

The readings of leaf chlorophyll content or greenness were taken with a handheld device Soil Plant Analysis Development (SPAD, Minolta Camera Co., Osaka, Japan) chlorophyll meter. Five readings were taken from newly and fully opened leaves of each plot with care so that the mid-rib of the leaf should not come under the eye of the instrument, and the average value was worked out.

The readings of spectral reflectance of leaves or NDVI values were taken by moving the instrument over the plot at DC37 and DC65 stages^20^ using a handheld optical sensor “GreenseekerTM handheld sensor” which measures the reflectance of a given crop area over a 61 cm × 61 cm area when the unit is positioned between 81 to 122 cm above the target area^21^. The NDVI is calculated from reflectance measurements in the red and near-infrared (NIR) portion of the spectrum.

$${\text{NDVI}} = \frac{{{\text{R}}_{{{\text{NIR}}}} {-}{\text{ R}}_{{{\text{Red}}}} }}{{{\text{R}}_{{{\text{NIR}}}} + {\text{ R}}_{{{\text{Red}}}} }},$$

where R_NIR_ is the reflectance of NIR radiation and R_Red_ is the reflectance of visible red radiation.

### Chemical analysis

Composite soil samples were collected prior to setting up the experiment layout, and subsequent to crop harvest, samples were collected from each plot at three depths (0–5 cm, 5–10 cm, and 10–15 cm). These samples were then processed and subjected to analysis to determine the available nitrogen content. Other chemical properties such as pH, EC, organic carbon, available P, and K were also analyzed, but no significant differences were observed among treatments and are presented as supplementary information.

Soil pH and EC were determined in a 1 and 2.5 ratio of soil and water suspension. The oxidizable organic C was determined using^22^ wet-oxidation method. An alkaline permanganate method (Subbiah and Asija, 1956) was followed to determine the KMnO_4_-oxidizable N. Olsen’s extractable P (soil pH > 6.0) was determined using the ascorbic acid reductant method^23^. Potassium (K) was extracted by neutral NH_4_OAc and detected using a flame photometer^23^.

Representative samples of crop produce (grain and straw) were collected randomly at harvest, dried, processed, and analyzed for nitrogen content. The amount of N removed by the crop was calculated by multiplying the percentage of nutrients with the yield of grain and straw separately and summing them up. The uptake of the crop was computed based on its dry weight as follows:

$${\text{Nutrient uptake }}\left( {{\text{kg}}/{\text{ha}}} \right){ = }\frac{{{\text{Nutrient content }}\left( \% \right) \, \times {\text{ Yield }}\left( {{\text{kg}}/{\text{ha}}} \right)}}{100}.$$

#### Agronomy efficiency (AE)

Nitrogen use efficiency is the yield increment per unit of N fertilizer given to the crop. It is measured in kg grain yield/kg nitrogen applied.

$${\text{Agronomic efficiency }}\left( \% \right) = \frac{{{\text{Yield}}_{{\text{fertilized plot}}} {-}{\text{ Yield}}_{{\text{control plot}}} }}{{{\text{F}}_{{{\text{applied}}}} }}.$$

Yield _fertilized plot_ and Yield _control plot_ are yields (kg/ha) when quantity of N fertilizer applied was F and zero; F_applied_ is the total nitrogen (kg/ha) applied^24^.

*Nitrogen use efficiency (NUE)* can be separated into two components: nitrogen uptake efficiency (NUpE) and nitrogen utilization efficiency (NUtE). NUpE describes the amount of nitrogen that a plant can take up from external sources, while NUtE describes the plant's ability to assimilate and remobilize nitrogen within its own tissues. NUE^25^ is the product of NUpE and NUtE^26,27^.

$${\text{N uptake efficiency}} \left( {{\text{NUpE}}} \right) \, = {\text{ Nitrogen contents in plant}}/{\text{Nitrogen supplied}},$$

$${\text{N utilization efficiency }}\left( {{\text{NUtE}}} \right) = {\text{Yield}}/{\text{Nitrogen contents in plant}},$$

$${\text{NUE}} = {\text{NUpE }} \times {\text{ NUtE}}.$$

#### Grain protein content or GPC (%)

The grain protein content of wheat was estimated by using Infratec™ 1241 (FOSS) instrument, a whole analyzer using near infrared (NIR) transmittance technology^25^.

### Economics

Wheat grain yield was multiplied by the minimum support price for 2021–22 (246.9 US $/ton) and 2022–23 (251.9 US $/ton) (reference for MSP from PIB, GOI). The wheat straw yield was also multiplied by the market rate (100 US $/ton) and added to get the gross return. The cost of cultivation was calculated by considering field preparation, seed, fertilizer, irrigation, transportation, herbicide application, the cost involved in harvesting and threshing of produce, management charges, the rental value of land, interest on fixed capital, depreciation cost of implements, and farm buildings. The net return was calculated by subtracting the cost of cultivation from gross returns. The benefit–cost ratio was calculated by dividing the gross return by the total cost of cultivation. To convert into US$, the gross return, cost of cultivation, and net return were divided by the prevailing exchange rate ($1 = Rs. 80).

### Partial budget analysis

A partial budget analysis was employed to assess the economic advantages of various nitrogen fertilizer application sources and timings in relation to wheat grain and straw yields. During this study, we identified the economically viable treatments by estimating their costs and benefits in accordance with the market prices for the specific research year. It's worth noting that experimental yields often surpass what farmers can realistically achieve when implementing the same treatments^28^. Gross and net benefits were computed using the following formulas:

(1) Gross returns = wheat grain and straw yield × Market price of respective yields,

(2) Net Benefit = Gross returns—Total variable cost^a^.

^a^Total variable cost was computed by deducting the fixed land cost of from total cost of cultivation.

### Marginal rate of return (MRR) and dominance analysis (DA)

The calculation of the marginal rate of return (MRR) involved dividing the change in net benefit by the change in variable cost, which represents the additional return gained by increasing the input. Before computing the MRR, a simplified dominance analysis, as outlined by Maize and Center in 1988^28^, was carried out to identify treatments that hold relevance for farmers in terms of earnings. A treatment is considered dominated when, despite incurring higher costs, it fails to yield a greater increase in net benefits. In such cases, it is dominated because there exists at least one other treatment with equal or lower costs that generates higher benefits. To conduct this analysis, treatments were arranged in ascending order of variable costs, and comparisons were made to determine whether an increase in costs was associated with a proportionally greater increase in net benefits.

### Statistical analysis

Analysis of variance (ANOVA) and ranking of treatments was completed using Tukey’s Range test at 0.05 (5%) level of significance. The General Linear Model (GLM) Procedure in SAS®9.3 version 6.1.7061 for Windows (Cary, NC, SAS Institute Inc., 2012) was used for statistical analysis.

### Ethics approval

All authors approve ethical responsibilities related with this manuscript.

## Results

### Effect on growth, yield attributes and yield parameters

**Figure 4:**
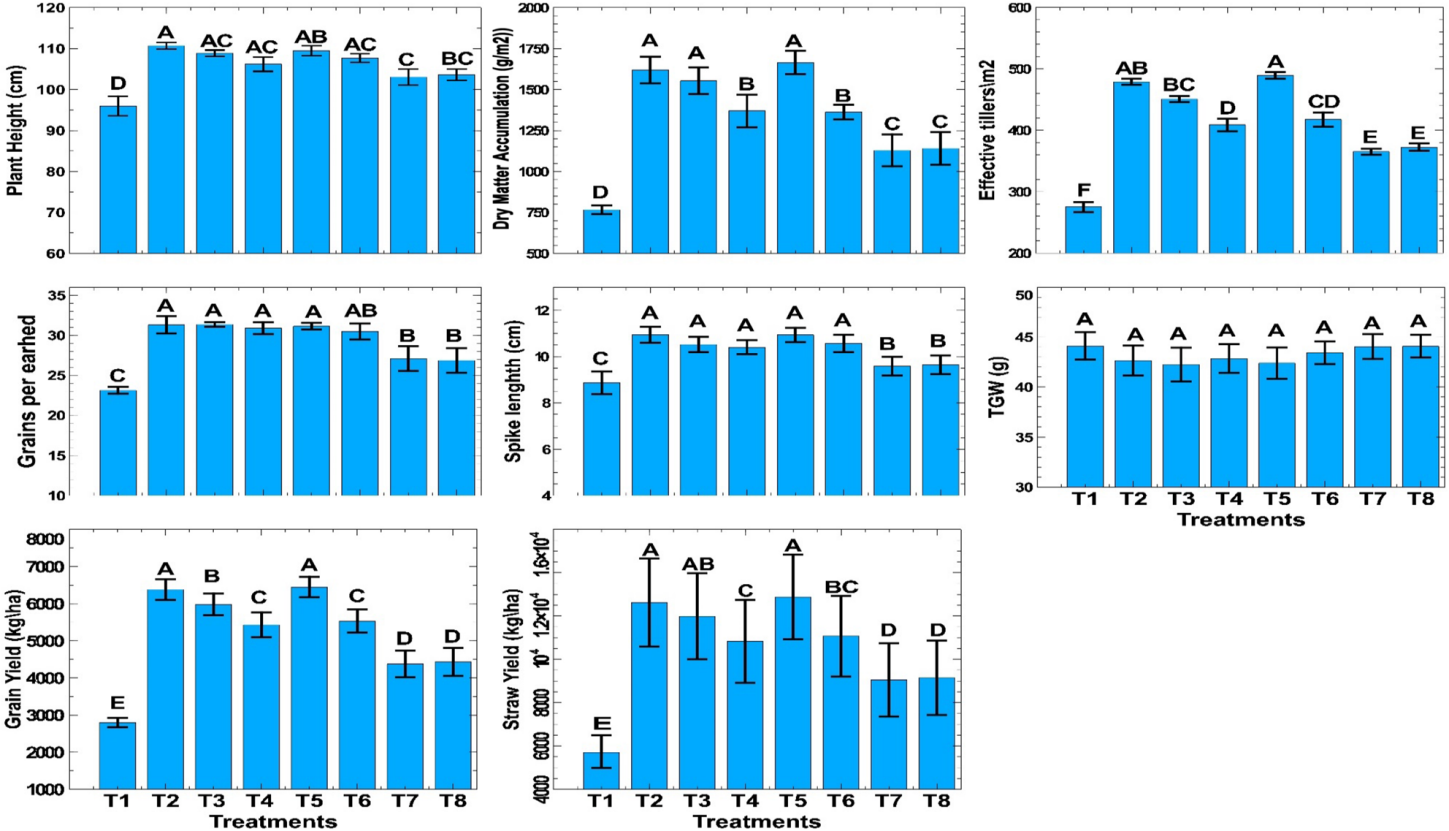
Effect of combined nitrogen sources on growth, yield attributes and yield of wheat under conservation tillage.

The Fig. 4 indicated the presence of significant effects of varying nitrogen applications through conventional and nano fertilizers on growth, yield attributes, and yield parameters of wheat. The application of 150 kg N/ha before irrigation resulted in increase in plant height which was statistically similar to the treatments where 100 kg N/ha or more was applied through conventional fertilizer (Urea) before (BI) or after irrigation (AI), as well as through nano urea (4% N). The dry matter accumulation under treatment with application of 150 kg N/ha BI + 1 spray of nano urea (T5) was non-significant as compared to 150 kg N/ha before (T2) and after irrigation (T3). However, this was significantly higher than dry matter accumulated in other treatments. It notable to mention that, treatments with spraying of either 1 or 2 additional sprays of Nano urea along with application of 100 kg N/ha could not compensate for the 50 kg N/ha supplied through conventional urea under farmer practice. This treatments recorded significantly lower dry matter content as compared to the conventional farmer practice (150 kg N/ha). The count of effective tillers (489.5) was significantly higher in treatment (T5) where 150 kg N/ha BI was applied along with one nano urea spray (4% N), which was non-significant with 150 kg N/ha BI (T2) (478.5). All growth parameters like plant height, dry matter accumulation, and effective tillers/m^2^ were significantly lowest in the control treatment compared to others. The number of grains per earhead and spike length were significantly higher in higher fertilized plots. Treatments with nitrogen doses of 100 or 150 kg/ha BI or AI with or without nano spray recorded significantly higher grains per earhead and spike length over other treatments. Zero nitrogen recorded the least number of grains per earhead (23.1) and spike length (8.87 cm). The thousand-grain weight did not vary significantly among treatments, and more values of thousand-grain weight were recorded in control plots.

Analysis of pooled data on grain yield (Fig. 4) revealed that the maximum grain yield (6448 kg/ha) was obtained with the application of 150 kg N/ha in three equal splits before irrigation (BI) with one nano urea spray (4% N) (T5). This treatment was statistically similar to the application of 150 kg N/ha in three equal splits BI alone (T2) (6380 kg/ha) but significantly higher than other treatments. The application of 150 kg N/ha in three splits before irrigation alone (T2) and along with one nano urea (4%) spray (T5) increased grain yield by 6.7% and 7.8%, respectively, compared to 150 kg N/ha after irrigation (T3). The application of 100 kg N/ha (BI) through conventional urea along with one or two sprays of nano urea (4% N) reduced grain yield by 14.9% and 13.3%, respectively, compared to the application of 150 kg N/ha before irrigation. Thus, the 1/3rd dose of nitrogen given through conventional urea cannot be supplemented by one or even two sprays of nano urea. The highest straw yield (9356 kg/ha) was obtained with the application of 150 kg N/ha in three equal splits BI with one nano urea spray (T5), which was at par with 150 kg N application in three equal splits before (T2) and after irrigation (T3).

### Effect on physiological parameters

**Figure 5:**
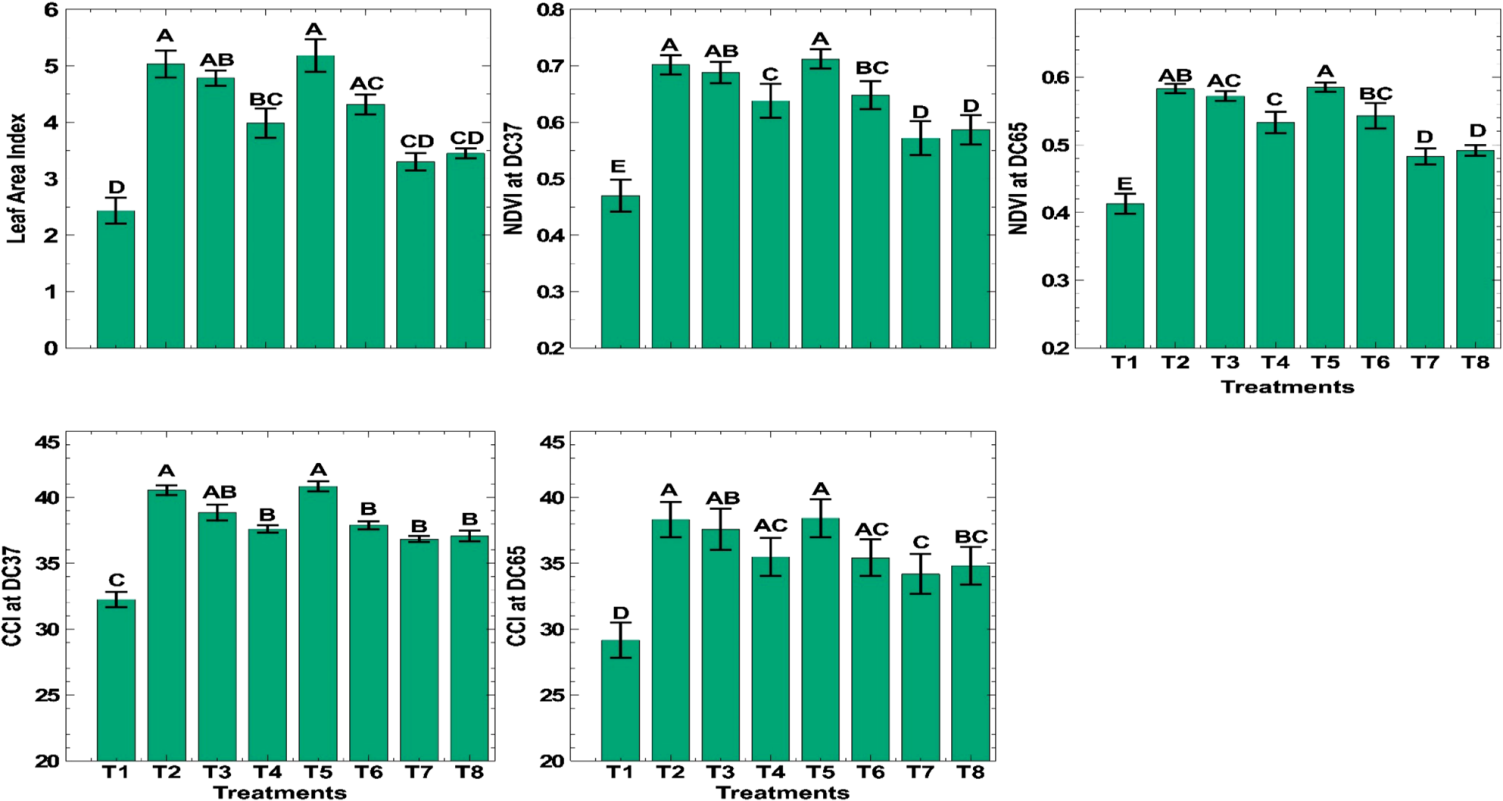
Effect of combined nitrogen sources on physiological parameters of wheat under conservation tillage.

Pooled data showed that the leaf area index at the maximum tillering stage was significantly higher when 150 kg N/ha was applied BI in three equal splits with one nano urea (4% N) spray (T5) (4.23), which was non-significant with 150 kg N application in three equal splits before (T2) (4.10) and after irrigation (T3) (3.95). The readings from Greenseeker and SPAD meters, such as NDVI and CCI at DC37 and DC65, varied significantly among treatments (Fig. 5). NDVI values at DC37 (0.71) were significantly higher in treatments where 150 kg N/ha BI + 1 nano urea spray (4% N) was applied, which was statistically similar to the treatment where 150 kg N/ha was applied before (0.70) and after irrigation (0.69). However, the corresponding values of NDVI at DC65 were decreased. Control plots recorded the lowest values of NDVI at both stages (DC37 and DC65). CCI at DC37 (40.9) was significantly higher in treatments where higher doses of nitrogen (150 kg N/ha or more) were applied compared to other treatments. At DC65, the CCI values decreased numerically from the corresponding treatments.

### Effect on economics

**Figure 6:**
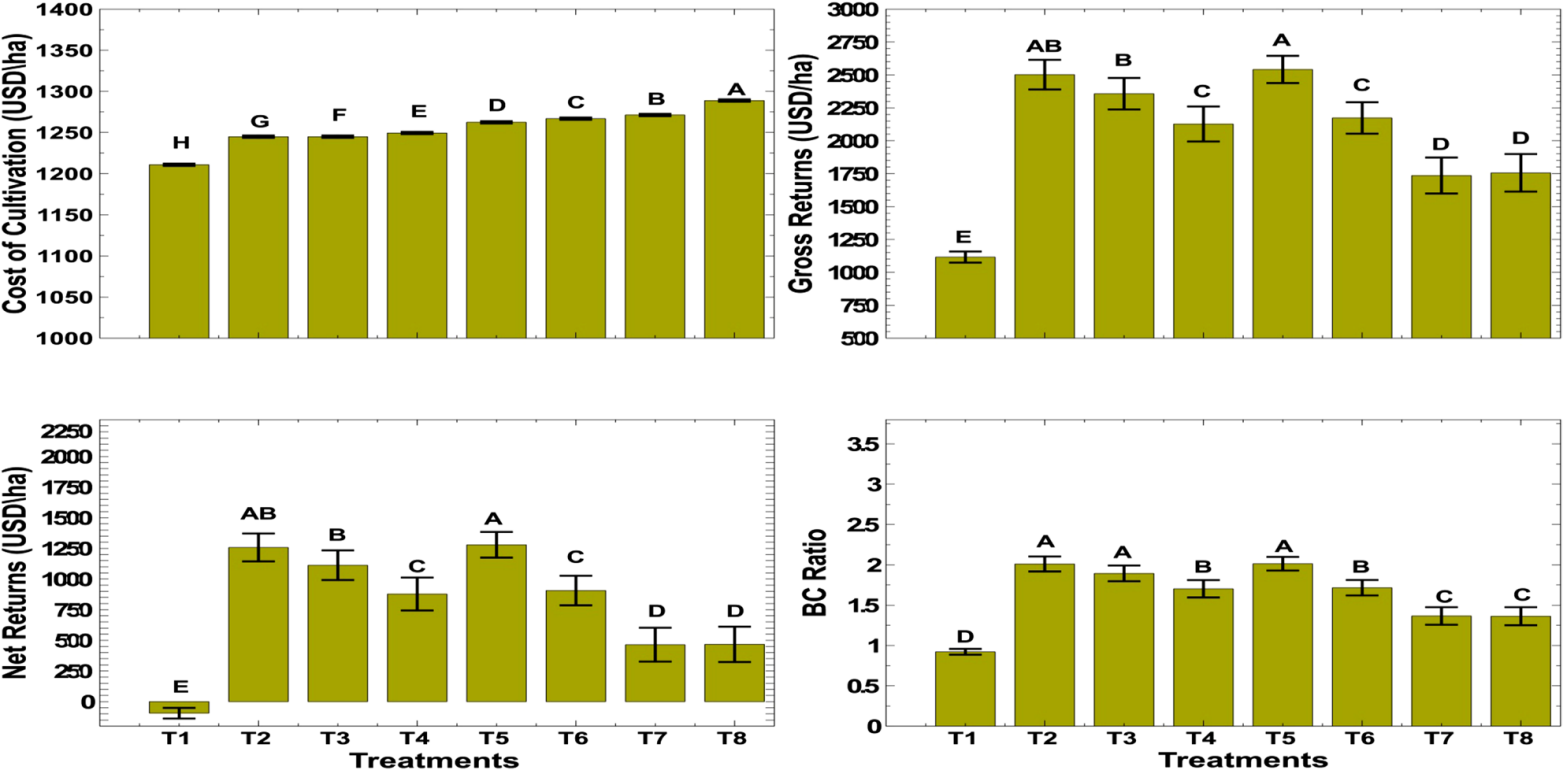
Effect of combined nitrogen sources on economics of wheat under conservation tillage.

The pooled data presented in Fig. 6 shows that the cost of cultivation increased with an increase in the number of inputs, i.e. conventional and nano urea nitrogen, and their application costs. The highest cost of cultivation was recorded in T8 (1289 US $/ha). The application of 150 kg N/ha in three equal splits before irrigation with one nano urea spray resulted in significantly higher gross returns (2542 US $/ha) and net returns (1279 US $/ha) as compared to other treatments, although it was non-significant with T2, which had gross returns (2503 US $/ha) and net returns (1257 US $/ha). The benefit–cost ratio (BC ratio) of T2 and T5 was same (2.0). The control plots recorded the least gross returns (1116 US $/ha), net returns (− 94.5 US $/ha), and BC ratio (0.92) reflecting loss.

The partial budget analysis given in Table 4 showed that the highest net benefits (2107.2 US $/ha) were recorded from the application of 150 kg N/ha in three equal splits as basal and just before 1st and 2nd irrigation along with one spray of nano urea (T5). Next to control (733.3 US $/ha), the net benefit was not in line with the increase in the cost of variables. It is highly affected by agronomic practices involved in the nitrogen management. The treatments T3, T4, T6 and T7 were dominated and can be eliminated for further consideration. These treatments have higher costs that vary but lower net benefits. The marginal rates of return for the non-dominated treatments with the application of 150 kg N/ha in three equal splits as basal and just before 1st and 2nd irrigation alone (T2) as well as along with one spray of nano urea (T5) were 3959 and 3077%, respectively. Therefore, these treatments were found to be economical viable options.

**Table 4:** Summary of the partial budget analysis for the use of different sources and timing of nitrogen fertilizer applications in wheat.

Treatment	TVC (US $)	NB (US $)	Cost increased	Benefit increased	DA	MRR (%)
T1	898.3	733.3				
T2	932.5	2085.4	34.2	1352.1		3959
T3	932.5	1940.4	0.0	– 145.0	D	
T4	936.9	1705.7	4.5	– 234.7	D	
T5	950.0	2107.2	13.1	401.5		3077
T6	954.4	1734.4	4.5	– 372.9	D	
T7	958.9	1292.2	4.4	– 442.1	D	
T8	976.4	1295.2	17.5	2.9		17

*TVC* total variable cost (US $/ha), *NB* net benefit (US $/ha), *DA* dominance analysis and *D* dominated, *MRR* marginal rate of return.

### Effect on soil properties and plant nutrient uptake

**Figure 7:**
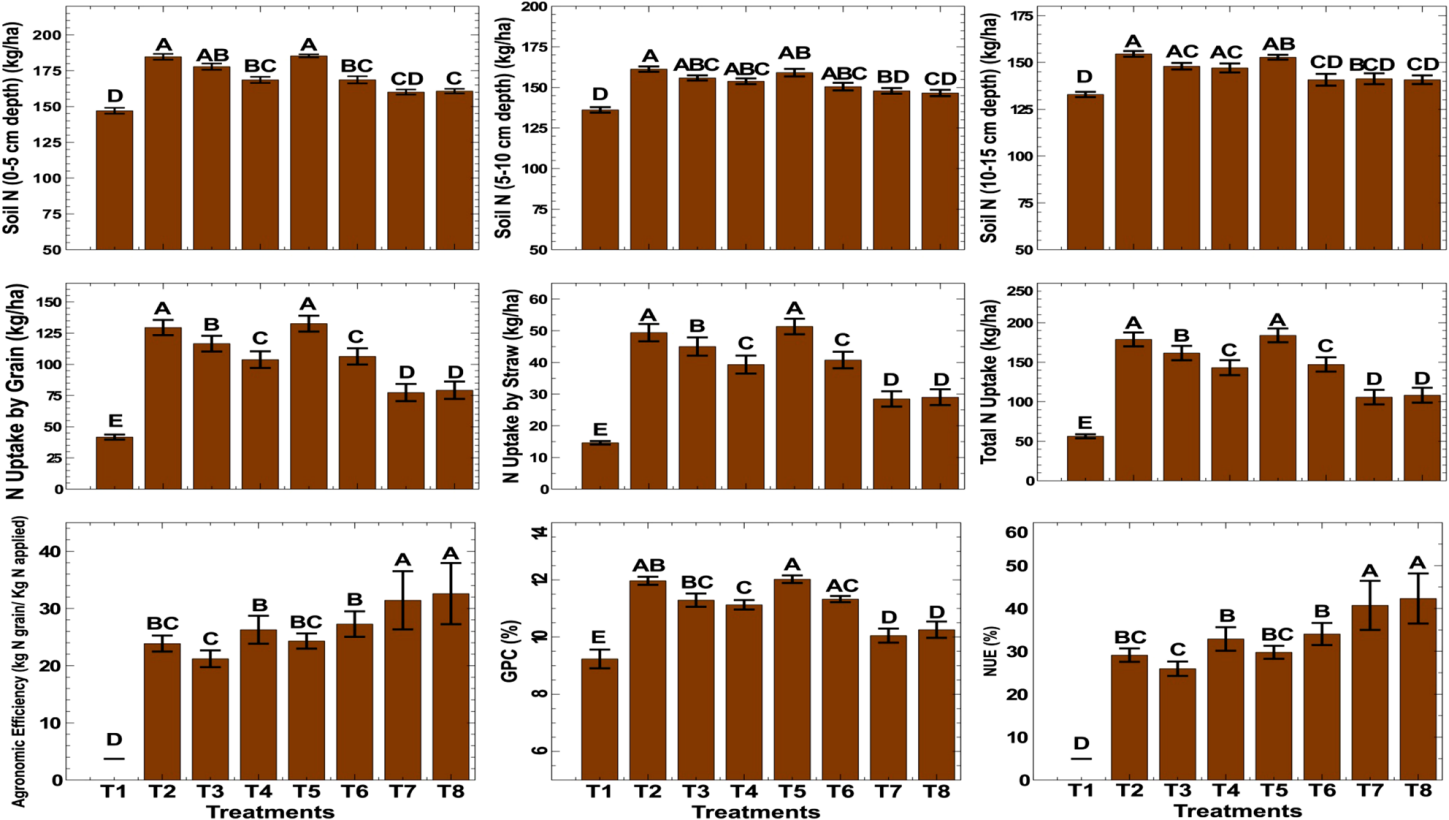
Effect of combined nitrogen sources on soil nitrogen status, nitrogen uptake, agronomic efficiency, NUE and GPC of wheat under conservation tillage.

The soil's available nitrogen status after the wheat harvest was found to be significantly different among treatments in all three layers (0–5, 5–10, and 10–15 cm), as shown in Fig. 7. The treatment that had 150 kg N/ha applied in three equal splits before irrigation alone or along with one spray of nano urea recorded significantly higher soil available nitrogen at the 0–5 cm depth, which was similar to the same dose of nitrogen applied after irrigation. The control treatment had the least available nitrogen after the harvest. The corresponding values of available nitrogen after the harvest decreased in deeper layers of 5–10 and 10–15 cm. However, the trend of soil nitrogen status in layers below 0–5 cm remained the same as that in the top layer. Other soil parameters such as pH, EC, OC, available phosphorus, and potassium analyzed after harvesting were not significantly different among treatments (Supplementary Table 1).

Nitrogen uptake by wheat grains, straw, and total uptake also varied significantly, as shown in Fig. 7 on a pooled basis. The application of 150 kg N/ha in three equal splits before irrigation with one spray of nano urea (T5) recorded significantly higher nitrogen uptake in grains (132.6 kg/ha), straw (51.3 kg/ha), and overall uptake (178.9 kg/ha), which was similar to 150 kg N/ha applied before irrigation in three equal splits (T2).

Agronomic efficiency (AE) was highest (32.6 kg grain yield/kg nitrogen applied) in the treatment where 50 kg N/ha was applied as basal and four sprays of nano urea (4% N) were applied (Fig. 7). It decreased as the dose of nitrogen increased. The full dose of N (BI) along with one spray of nano urea showed an AE of 26.3 kg grain yield/kg nitrogen applied. NUE also followed the similar trend of results as AE. The application of 150 kg N/ha in three equal splits before irrigation with one spray of nano urea (T5) recorded significantly higher grain protein content (GPC) of 12.02%, which was similar to the application of 150 kg N/ha before irrigation in three equal splits (T2) with a GPC of 11.97%.

### Correlation between physiological parameters and grain yield

**Figure 8:**
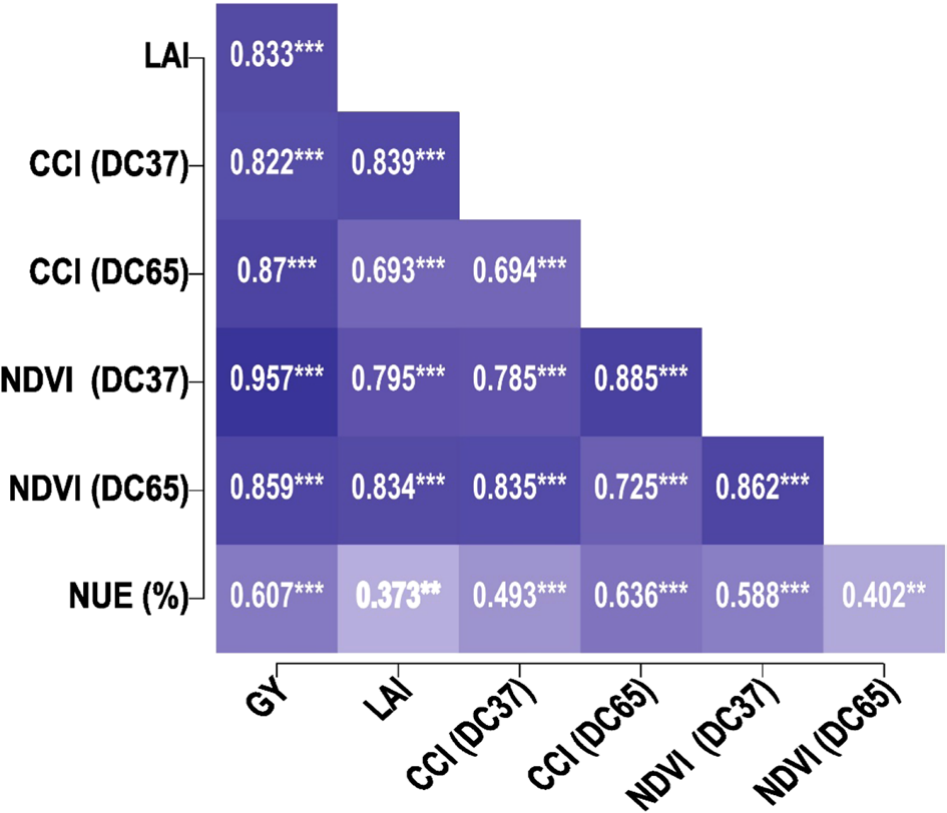
Simple correlation matrix of grain yield and physiological parameters.

The correlation coefficients among grain yield and other parameters were significant and highly positive, as shown in Fig. 8. The correlation coefficient between grain yield and LAI was 0.833, between grain yield and CCI at DC37 was 0.822, between grain yield and CCI at DC65 was 0.870, and between grain yield and NDVI at DC37 was 0.957 and at DC65 was 0.859. The correlation between grain yield and AE was also significant but not highly positive, as it varied with different doses of nitrogen supplied through conventional urea and nano urea.

## Discussion

Nitrogen is one of the most crucial plant nutrients that affects the growth and development of wheat^29^. However, the application of conventional urea (46% N) on the soil surface leads to significant losses ranging from 1 to 60% in the form of ammonia, volatilization, and nitrous oxide emissions^30–32^. The increase in growth parameters of wheat is linked to a sufficient supply of nitrogen that contributes to increased enzymatic activity^30^. The sandy loam texture and poor fertility condition of soils can lead to improved wheat growth characteristics and yield with nitrogen fertilization^33^. Increasing the nitrogen dose up to a certain level enhances the concentration of nitrogen in the leaves, thereby boosting the plant's capacity for photosynthesis and resulting in an increase in grain yield^34,35^. The use of nano fertilizers offers a solution to the problem of nitrogen volatilization, as they have variable solubility, effectiveness, high consistency, time-controlled discharge, enhanced targeted activity, and effective concentration^19^. Foliar application of nano-fertilizers can enter plants through the epidermis or stomata and then translocate through the apoplast or symplast pathways, which enhances nitrogen use efficiency^36,37^. However, it is essential to use a proper concentration of nano fertilizers to meet the crop’s nutrient requirements. Lower concentrated nano fertilizers could be used as a supplementary source to enhance quality without any significant loss in yields and profits. In our study, the foliar sprays of nano urea in addition to 150 kg N/ha applied through prilled urea enhanced grain yield, however the increase was not significant compared to a similar dose of nitrogen applied through urea. This lack of significance might be attributed to the lower concentration of nitrogen in nano urea, which did not effectively improve the yield attributes. Similar results have been reported by Rawate et al.^38^ and Saklani and Pal^39^. These results are in contradiction with Al-Juthery et al.^40^**,** Mehta and Bharat^41^, Fakharzadeh et al.^42^ and Poudel et al.^43^ which might be due to different nature of nanofertilizers (NFs) used, varying concentrations of NFs, prevailing conditions, timing and method of application, etc. A research paper recently published by Frank and Husted^44^ created controversy by challenging the manufacturer's claims regarding the poorly description of the product with no scientifically proven effects. In our study, we found that there are some effects of nano-urea on the growth and yield of wheat but cannot replace a bag of urea (45 kg urea or 20.7 kg of N) as claimed by product manufacturers. However, a recent research has supported two foliar sprays of nano-urea reduced the nitrogen load by 25% without negatively impacting crop yield of wheat, maize, mustard and pearl millet^45^.

Applying nitrogen at a rate of 150 kg/ha with urea top dressing just before irrigation increased grain yield by 5.7–7.9% as compared to urea top dressing after irrigation. This improvement can be attributed to the percolation of urea with irrigation water below surface immediately after top dressing thus protect the hydrolysis products from volatilization, results in to nitrogen storage to the root zone of the crop, particularly in sandy loam soil. This method reduced the nitrogen losses through ammonia volatilization under conservation tillage^46–48^.

In summary, the application of nitrogen is critical for the growth and development of wheat. The use of nano fertilizers can enhance nitrogen use efficiency and reduce losses due to nitrogen volatilization. Therefore, it is necessary to use a proper concentration of nano fertilizers to meet the crop’s nutrient requirements, while lower concentrations can be used as a supplementary source to enhance quality without significant losses in yields and profits.

Optimizing nitrogen management in conservation tillage requires careful timing of nitrogen application. Urea, which is rapidly hydrolyzed to ammonium, is susceptible to losses through ammonia volatilization. These losses can be reduced by either applying urea 1–2 cm below the soil surface or altering the timing of urea application (before or after irrigation). In coarse-textured soils with high water percolation rates, urea application should be done before irrigation to allow percolating water to carry urea to the root zone, minimizing the potential for ammonia volatilization. Studies have shown that applying nitrogen before irrigation moves urea to the 15–30 cm layer, which is later moved back to the soil surface with capillary rise due to water evaporation, making it more available to crop plants^47–49^. Therefore, in conservation tillage, nitrogen should be applied before irrigation to maximize productivity and profitability. However, from an economic standpoint, complete replacement of conventional fertilizers with nano fertilizers is not economical. Instead, supplying nutrients in combination (conventional + nano) has been shown to have a significant effect in increasing net returns^50^. Therefore, it is essential to manufacture nano fertilizers with the appropriate concentration to ensure their cost-effectiveness.

Positive correlations were observed between nitrogen application rates and NDVI and CCI values in wheat. As the dose of nitrogen increased, its uptake intensified chlorophyll content and resulted in higher grain yield^51,52^. Similarly, CCI and NDVI values were positively correlated with nitrogen application, with an increase in wheat greenness, leaf area index, and vigorous growth observed as nitrogen application rates increased from zero to maximum^53,54^.

Conservation tillage practices, which involve retaining leftovers from previous crops, can improve agro-ecosystems under the rice–wheat cropping system by holding moisture, controlling weeds, and gradually supplying nutrients through decomposition^5,55^. The retention of rice residue in the wheat crop was found to boost growth and production compared to no residue, attributed to the gradual provision of nutrients from rice remaining in the soil and moisture retention in the rhizosphere during increasing minimum temperature on later stages. Crop leftovers decay slowly over the course of the growing season, releasing nutrients that improved plant growth and increased yield. Conservation tillage was observed to increase wheat grain yield by 10–20% compared to conventional tillage^56,57^, which can be attributed to improved soil health and maintained soil moisture in the root zone, providing a better environment for wheat growth and development. An important advantage of conservation tillage is the reduction of production costs by US $33 to 50^58–60^, including the need for labor and fuel used in tillage operations and equipment maintenance. Among conservation tillage practices, zero tillage for wheat production was found to be most successful in terms of good crop establishment and increased yield^61–63^. The treatment with the highest gross and net returns was T5, directly proportional to the yield of grain and straw. Top dressing of urea just before irrigation resulted in higher gross returns and net returns over top dressing after irrigation owing to more grain and straw yield obtained in the former treatment at similar costs of cultivation. The profitability of these treatments is applicable only for the current agricultural year under investigation. This assessment will need to be conducted again in subsequent agricultural years because of natural fluctuations in prices.

## Conclusions

After 2 years of study, it can be concluded that applying 150 kg N/ha as basal and remaining 2/3rd just before 1st and 2nd irrigation in three equal splits under conservation agriculture, along with or without a single spray of nano urea (4% N), leads to better growth, yield attributes, and yield of wheat. The highest marginal rate of return recorded with the application of 150 kg N/ha as basal and remaining 2/3rd just before 1st and 2nd irrigation in three equal splits. The additional spray of nano urea has gained the net benefit but the MRR was further reduced. This application also enhanced the soil nitrogen status, nitrogen uptake by wheat, and grain protein content. But the additional spray of nano urea has not enhanced these parameters significantly. As claimed, nano urea cannot substitute the one bag *i.e.,* 45 kg of prilled urea (46% N). The combined use of conventional urea (46% N) and nano urea (4%) can help in enhancing agronomic efficiencye, but there is a need to work on the concentration of nano urea, which is currently low compared to the requirement of wheat for proper physiological functioning.

Under Indian conditions, farmers need to shift from their current practice of applying N through urea 7–10 days after irrigation to before irrigation to achieve maximum wheat productivity, profitability, and agronomic efficiency. However, after N application, farmers must ensure irrigation under dry residue retention conditions, as otherwise, most of the N will be lost through volatilization. Nitrogen applied after irrigation has less nitrogen use efficiency as compared to nitrogen applied before irrigation. It can further be enhanced though nano urea but it can’t substitute the prilled urea due to lower concentration of nitrogen (4% N) particularly in wheat. However, further comprehensive research is required for the use of nano urea at higher concentrations by taking phytotoxicity into consideration.

### Supplementary Information

Supplementary Table 1.

## Data Availability

All the data will be available on request to corresponding author.
