# Supplementary material for: Boosting wheat yield, profitability and NUE with prilled and nano urea in conservation tillage
Source: Sci Rep. 2023 Oct 23;13:18073. doi: 10.1038/s41598-023-44879-w (PMC10593831; doi:10.1038/s41598-023-44879-w)
Supplement: Supplementary file 1 — Supplementary Table 1. [file 41598_2023_44879_MOESM1_ESM.docx]

**Table 1: Effect of combined application of conventional and nano urea on soil properties of wheat under conservation tillage**

| Treatment | pH | | | | | | EC (dS/m) | | | | | | Organic carbon (%) | | | | | | Available phosphorus (kg/ha) | | | Available potassium (kg/ha) | | |
| --- | --- | --- | --- | --- | --- | --- | --- | --- | --- | --- | --- | --- | --- | --- | --- | --- | --- | --- | --- | --- | --- | --- | --- | --- |
|  | 0-5 cm | | 5-10 cm | | 10-15 cm | | 0-5 cm | | 5-10 cm | | 10-15 cm | | 0-5 cm | | 5-10 cm | | 10-15 cm | | 0-5 cm | 5-10 cm | 10-15 cm | 0-5 cm | 5-10 cm | 10-15 cm |
|  | 2020-21 | 2021-22 | 2020-21 | 2021-22 | 2020-21 | 2021-22 | 2020-21 | 2021-22 | 2020-21 | 2021-22 | 2020-21 | 2021-22 | 2020-21 | 2021-22 | 2020-21 | 2021-22 | 2020-21 | 2021-22 | 2020-21 | 2021-22 | 2020-21 | 2021-22 | 2020-21 | 2021-22 |
| T_1_ | 7.86 | 7.85 | 7.94 | 7.97 | 8.04 | 8.08 | 0.161 | 0.163 | 0.150 | 0.145 | 0.140 | 0.138 | 0.63 | 0.60 | 0.62 | 0.59 | 0.56 | 0.54 | 20.1 | 20.3 | 16.6 | 17.1 | 15.5 | 15.4 |
| T_2_ | 7.78 | 7.77 | 7.91 | 7.91 | 8.06 | 8.03 | 0.176 | 0.175 | 0.144 | 0.148 | 0.137 | 0.139 | 0.68 | 0.66 | 0.63 | 0.63 | 0.60 | 0.59 | 19.1 | 18.8 | 16.2 | 16.2 | 15.5 | 15.1 |
| T_3_ | 7.59 | 7.77 | 7.91 | 7.92 | 8.00 | 8.11 | 0.182 | 0.165 | 0.160 | 0.148 | 0.141 | 0.133 | 0.65 | 0.63 | 0.60 | 0.58 | 0.54 | 0.58 | 19.2 | 19.0 | 16.7 | 16.2 | 15.5 | 14.7 |
| T_4_ | 7.72 | 7.74 | 7.81 | 7.90 | 7.97 | 8.01 | 0.172 | 0.178 | 0.143 | 0.148 | 0.139 | 0.127 | 0.64 | 0.62 | 0.64 | 0.62 | 0.59 | 0.59 | 19.3 | 19.0 | 16.6 | 16.6 | 15.5 | 15.9 |
| T_5_ | 7.83 | 7.80 | 7.99 | 7.98 | 8.10 | 8.09 | 0.180 | 0.167 | 0.148 | 0.143 | 0.131 | 0.134 | 0.67 | 0.66 | 0.61 | 0.62 | 0.59 | 0.59 | 19.2 | 18.8 | 16.3 | 16.5 | 15.2 | 14.8 |
| T_6_ | 7.84 | 7.80 | 8.01 | 7.99 | 8.10 | 8.10 | 0.173 | 0.177 | 0.145 | 0.144 | 0.136 | 0.133 | 0.67 | 0.65 | 0.64 | 0.62 | 0.58 | 0.58 | 19.0 | 18.8 | 16.5 | 16.1 | 15.4 | 15.2 |
| T_7_ | 7.85 | 7.86 | 7.97 | 7.96 | 8.09 | 8.10 | 0.156 | 0.171 | 0.131 | 0.139 | 0.120 | 0.129 | 0.65 | 0.63 | 0.63 | 0.62 | 0.60 | 0.58 | 19.2 | 19.1 | 16.7 | 16.6 | 15.6 | 15.8 |
| T_8_ | 7.73 | 7.91 | 7.88 | 8.01 | 8.03 | 8.11 | 0.165 | 0.170 | 0.154 | 0.143 | 0.150 | 0.139 | 0.65 | 0.64 | 0.60 | 0.60 | 0.57 | 0.57 | 19.4 | 19.2 | 16.6 | 16.9 | 15.6 | 15.6 |
| SE (m) ± | 0.07 | 0.06 | 0.04 | 0.05 | 0.05 | 0.05 | 0.016 | 0.004 | 0.009 | 0.006 | 0.007 | 0.004 | 0.02 | 0.02 | 0.04 | 0.02 | 0.02 | 0.02 | 0.8 | 0.7 | 0.6 | 0.8 | 0.5 | 0.7 |
| CD (p=0.05) | NS | NS | NS | NS | NS | NS | NS | NS | NS | NS | NS | NS | NS | NS | NS | NS | NS | NS | NS | NS | NS | NS | NS | NS |
